# Looking for the elusive lung stem cell niche

**DOI:** 10.1186/2213-0802-2-7

**Published:** 2014-04-03

**Authors:** Ena Ray Banerjee

**Affiliations:** Department of Zoology, Immunology and Regenerative Medicine Research Laboratory, University of Calcutta, 35, Ballygunge Circular Road, Kolkata, 700019 West Bengal India

**Keywords:** Stem cells, Lung progenitors, Lung stem cell niche, BrdU pulse chase assay, Side population cells

## Abstract

This discourse contains three perspectives on various aspects of Stem Cell Biology and tools available to study and translate into Regenerative Medicine. The lung incessantly faces onslaught of the environment, constantly undergoes oxidative stress, and is an important organ of detoxification. In degenerative diseases and inflammation, the lung undergoes irreversible remodeling that is difficult to therapeutically address and/or transplant a dying tissue. The other difficulty is to study its development and regenerative aspects to best address the aforementioned problems. This perspective therefore addresses- firstly, review of types of stem cells, their pathway of action and models in invertebrate organisms vis-a-vis microenvironment and its dynamics; secondly, stem cells in higher organisms and niche; and lastly data and inference on a novel approach to study stem cell destruction patterns in an injury model and information on putative lung stem cell niche. Stem cells are cryptic cells known to retain certain primitive characteristics making them akin to ancient cells of invertebrates, developmental stages in invertebrates and vertebrates and pliant cells of complex creatures like mammals that demonstrate stimulus-specific behavious, whether to clonally propagate or to remain well protected and hidden within specialized niches, or mobilize and differentiate into mature functionally operative cells to house-keep, repair injury or make new tissues. In lung fibrosis, alveolar epithelium degenerates progressively. In keeping with the goal of regenerative medicine, various models and assays to evaluate long and short term identity of stem cells and their niches is the subject of this perspective. We also report identification and characterization of functional lung stem cells to clarify how stem cell niches counteract this degenerative process. Inferences drawn from this injury model of lung degeneration using a short term assay by tracking side population cells and a long term assay tracking label retaining cells have been presented.

## Perspective 1. A mini review on stem cells, their pathway of action and stem cell models

### Introduction

As defined by researchers stem cells are cells that have the ability of self renewal through cell division and differentiate into a diverse array of cell lines [1].

In the general sense, stem cells need to fulfill the following four criteria to be classed as stem cells; a. in order to maintain the stem cell population, stem cells should be capable of continuous self renewal b. stem cell should have the ability to differentiate into a variety of mature cells c. stem cells should be able to integrate and differentiate into its source damaged site d. lastly, stem cells should have the ability to differentiate into mature cells of a tissue even if the tissue doesn’t suffer [2].

With recent developments in the field and advancement of technologies like fluorescence activated cell sorting (FACS), magnetic activated cell sorting (MACS) along with enhanced isolation, culture and molecular imaging techniques [2] there has been much speculation of its use in therapy, regenerative medicine, drug and toxicity screening [1]. Essentially, cell based therapy or regenerative medicine is a three dimensional operation which includes involvement of researchers/clinicians and companies in a particular cell therapy, types of cells (autologous and allogenic) and the subsequent scale of manufacture and finally integration of cellular therapy with clinical practice [3].

Tackling degenerative disorders in the ever ageing human population is one of the biggest challenges faced by clinicians today with surgeries and drugs being the gold standard for treatment. Stem cells have been proven to have the ability to maintain and replenish tissues and therefore, stem cells or stem cells coupled with gene therapy can be used as potential means for treating degenerative disorders and restore tissue function.

Tissue restoration can be accomplished either through stem cell integration directly in the damaged/target tissues or by delivering complex signals to target tissues without any integration. For e.g. hematopoietic stem cells (HSCs) can restore tissue function by directly integrating into the target tissue while mesenchymal stem cells (MSCs) tend to deliver the signals to target tissues like in ischemic cardiac injury. Infusion of MSCs has also shown therapeutic use in amelioration of symptoms in bleomycin – induced mouse lung injury. However, owing to lack of absolute evidence and experimental works still being carried out on the immunological and tissue trophic effects of MSCs, their therapeutic potential remains unclear. On the other hand HSCs show engraftment into bone marrow during development as shown by the expression of CXC chemokine, stromal derived factor – 1(SDF – 1), although, SDF-1 and its receptor CXCR4 are not essential for bone marrow engraftment, thus, desiring lot more work to be carried out on localization and engraftment of stem cells before clinical application is accomplished.

As a result of these barriers, recapitulating tissue structures is being used as an alternative for e.g. in this case vascular cells derived from human embryonic stem cells (ESCs) have the ability to amass in to blood carrying conduits (*in vivo*) and spontaneously perform anastamoses with the host vasculature, thereby, indicating intrinsic morphogenetic assistance for cell based therapy. In addition to this bioengineered scaffolds will enhance and hasten the process of regenerative therapy for e.g. cardiomyocytes assemble into functional units on biocompatible thin films *in vitro* that can co – ordinate synchronous impulse propagation and can be shaped into 3D structures.

Preceding localization and engraftment of stem cells we need to realize the implications of immune barriers on stem cell transplants. In terms of immunity stem cells can be classified as autologous (taken from patient tissues) which doesn’t spark an immune reaction and allogenic (taken from unrelated donors) which may spark immune reaction requiring immune suppression. In order to circumvent the problems of immune suppression in regenerative therapy researchers have come up with genetically equivalent (isogenic) cells. These isogenic cells are produced by somatic cell nuclear transfer (SCNT; wherein adult somatic cell nucleus is injected into an enucleated oocyte) forming pluripotent ESCs from adult somatic cells. Alternatively, this is achieved by reprogramming adult somatic cells back to a pluripotent state using a set of transcription factors, also known as induced pluripotent stem cells (iPS cells).

N.B. ESCs produced by SCNT have only been done on animals and not humans yet [4].

### Stem cell in regenerative therapy

Apart from HSCs having been used as therapy for leukemia and other types of cancer, there has been a drastic increase in the use of MSCs as potential treatment for bone and cartilage repair, spinal cord injury, lung fibrosis, cardiovascular repair etc.

Examples: Orlic *et al*. [5] through his works showed locally delivered bone marrow cells could regenerate myocardium, indicating stem cell therapy could be useful for treating coronary artery disease. Gussoni *et al*. [6] showed that murine MSCs could be a potential tool for treating muscular dystrophy as the MSCs expressed dystrophin in conjunction with the sarcolemma when injected into the quadriceps muscle of *mdx* mice [7].

### Stem cells in drug and toxicity screening

Pathological modeling and drug screening using stem cells more specifically human pluripotent stem cells holds exciting and promising opportunities to identify new therapeutic approaches. Pluripotent stem cells can be used in screening to identify and evaluate the effects of compounds on specific human cell types which are predisposed to potential toxicity. Firstly, this process involves differentiation of human pluripotent stem cells into cells of a desired tissue that we wish to inspect using the investigative drug, which can then facilitate the study of dose – response toxicity analysis. Till date most such studies have been carried out on human pluripotent stem cell derived cardiomyocytes and hepatocytes. More recently such drug screening studies have been carried out on human pluripotent stem cell derived neurons to check for drug metabolism and to assess cellular toxicity. However, the one question that still needs to be addressed is whether such drug – toxicity studies on human pluripotent stem cell systems corroborate with results observed in the complex *in vivo* environment [8].

Finally, it must be noted that in order to fully exploit the different forms of stem cells we need a better understanding of organ morphogenesis. Further developments in developmental biology together with stem cell biology and tissue engineering hold the promise to ultimately transform regenerative medicine [9].

#### Classification of stem cells based on their differentiation potential

Based on differentiation potential stem cells can be classified into 5 groups namely, totipotent/omnipotent, pluripotent, multipotent, oligopotent and unipotent [1].

##### Totipotent/omnipotent

Cells with the ability to differentiate into embryonic and extra-embryonic tissues and form a complete viable organism are called totipotent. E.g. zygote.

##### Pluripotent

Cells with self – renewal capacity and ability to differentiate into the ectoderm, mesoderm and endoderm are classed as pluripotent. These cells are highly useful for regenerative medicine. E.g. ESCs and iPS cells.

##### Multipotent

Cells having the ability to differentiate to a limited number of cell fates or into closely related family of cells are termed multipotent. Unspecialized mesodermal MSCs having the ability to differentiate into connective tissues, bone, cartilage, circulatory and lymphatic systems are an example of multipotent cells.

##### Oligopotent

Oligopotent progenitor cells have the ability to differentiate into only a few closely related cell types. Lymphoid or myeloid stem cells are examples of oligopotent cells. These cells can form various blood cells like B and T cells but not a different blood cell type like red blood corpuscles.

##### Unipotent

These cells can differentiate into only one cell type and have least potency amongst stem cells. E.g. Muscle stem cells [1].

#### Classification of stem cells based on origin and their sources

Based on origin stem cells can be categorized into embryonic, fetal, perinatal, adult and iPS [1].

### Embryonic stem cells (ESCs)

ESCs are derived from the blastocyst (stage of embryo formed 5 – 6 days after fertilization). The blastocyst comprises of the inner cell mass (ICM) and the trophoblast which form the embryo and the placenta respectively. The ESC lines are derived by separating the ICM from the trophoblast. The ICM is then transferred into cell plates where under specific conditions these cells can be maintained and propagated infinitely in an undifferentiated state. ESCs just like any other cell forms show genetic instability and the addition or removal of a growth factor or precursor may initiate differentiation.

### Fetal stem cells (FSCs)

Fetal stem cells are sourced from embryos of terminated pregnancies. Although, not as potent as ESCs and unable to divide indefinitely in culture, FSCs have been used to produce neural stem cell lines some of which are already facing clinical trials in USA and U.K.

### Perinatal stem cells

Perinatal stem cells can be classed into 3 groups based on their origin; amniotic fluid stem cells, placental stem cells and umbilical cord stem cells.

### Amniotic fluid

Amniotic fluid stem cells are obtained from the amniotic fluid by amniocentesis (which results in ~1% chance of miscarriage) from 4th week onwards as the embryo is surrounded by the amniotic fluid at this point. The amniotic fluid is rich fetal epithelial cells which have shown characteristics similar to MSCs. Having gained popularity as a potential tool for regenerative therapy these cells are being widely cryopreserved.

### Placenta

Stem cells care collected from the placenta at the end of pregnancy (terminal placenta). Stem cells isolated from the amnion and placental villi show characteristics of both MSC and HSC progenitors. Placental blood is also a rich reservoir of stem cells. Placental stem cells do not divide indefinitely *in vitro*. Currently, clinical trials are being conducted to determine its use for therapy in limb ischemia.

### Umbilical cord

Umbilical cord blood collected from the umbilical cord after childbirth is rich in stem cells and has use in treating hematopoietic system disorders. However, due to the limited yield of cord blood stem cells and their limited expansibility it is quite difficult to treat anyone above the age range of 5 – 7 years. As a result private cord blood stem cell banks are also preserving placental blood along with cord blood to increase the quantity of stem cells. Recent research has shown Wharton’s Jelly (present within the umbilical cord and composed of mucopolysaccharides) to house fibroblasts and macrophages. These fibroblasts have been implicated to have stem cell potential.

### Adult stem cells (ASCs)

Adult stem cells are tissue restricted undifferentiated cells that multiply by cell division to replenish dying cells and regenerate damaged tissues. For e.g. human epidermis gets renewed every 3 – 4 weeks. Adult stem cells can be multipotent or oligopotent or unipotent depending on its specificity. Although, *in vitro* proliferation of these cells is very limited, researchers across the world are trying to figure out ways to stimulate these cells for differentiation into different cell types to be used for repairing tissue damage (regenerative therapy). ASCs can be easily isolated form bone marrow, adipose tissues and peripheral blood. Clinical trials across the world are being conducted on ASCs with variable success.

### Bone marrow

The success story of bone marrow stem cells has been in the successful transplant of bone marrow in the treatment of hematopoietic disorders as it is the safest form of cell therapy. More recently autologous bone marrow stem cells are being used to treat neurological disorders (e.g. multiple sclerosis, amyotrophic lateral sclerosis, Alzheimer’s), arthritis, heart and eye disorders, muscular degeneration, diabetes mellitus -1, 2 etc.

### Adipose tissue

In comparison to other tissues, adipose tissues house more stem cells. Stem cell markers are expressed in ~ 50% of nucleated non – fat cells extracted through liposuction. Although, clinical trials are in progress the major problem with adipose derived stem cells is to extract sufficient quantity of cells for therapy, also, only ~2% of cells infused cell for gets engrafted in organs [1].

### Peripheral blood

peripheral blood stem cells (PBSCs) can be collected from peripheral circulation (requires mobilization with hematopoietic growth factors). PBSCs cells are now being increasingly used as a source of allogenic transplants in emergency cases. However, we still need a better understanding of immunological reactions (graft – versus – host disease or GVHD) in PBSC transplants [10].

### List of adult stem cells

Hematopoietic stem cell, bone marrow stromal cells (MSCs), neural stem cells, epithelial stem cells (http://stemcells.nih.gov/staticresources/info/basics/StemCellBasics.pdf - page11).

#### iPS cells

In 2006 Shinya Yamanaka’s team at Kyoto University, Japan created the first iPS cell lines from mouse fibroblasts by introducing the following four factors; Oct 3/4, Sox2, c – Myc and Klf4. This was then followed up by human iPS cells in 2007. These cells have the ability to differentiate indefinitely *in vitro* with the capability of forming any mature human cell type and therefore, can be used to dispel immunosuppressants completely in case of patients receiving iPS cell treatment. Unfortunately, there are still a few doubts in the use of iPS cell with some of them being, a safe delivery method for iPS cell precursors without severely effecting the genome, xeno – free culture condition etc. [1].

### Pathways of stem cell activity

#### Stem cell niche

Stem cell niche are specific anatomical region within a particular tissue which houses the stem cell population. This site regulates tissue regeneration, repair and maintenance and protects stem cell from depletion and the host tissue from over proliferation, thereby, maintaining tissue physiology in organisms. It may be mentioned here that an abnormal niche activity can result in healthcare disorders e.g. cancer. Understanding the ‘niche’ therefore, is important to improve regenerative medicine.

Essentially there are 3 factors governing niche function and maintenance, namely, the extracellular matrix, paracrine factors and metabolism. An example of the effect of extracellular matrix includes the expression of osteopontin (OPN) a matrix protein. OPN deficiency in animals shows an increased HSC number which is dependent on the stem cell microenvironment. Paracrine factors have been implicated in niche activity too, for example unpaired (UPD) is produced by niche cells that regulate stem cell renewal through JAK – STAT signaling in drosophila testis. And lastly, an example of the effect of metabolism on stem cell niche is the site active bone remodeling where there is very high concentration of calcium levels which modulates osteoclast and osteoblast activity.

Finally, it may be said that a better understanding of the stem cell niche and its manipulation can aid regenerative therapy and can be used as a target site to treat cancer or at least limit the malignancy of cancer stem cells [11].

#### Stem cell mobilization and homing

Migration of HSCs from the bone marrow into the blood is termed stem cell mobilization. Mobilization of HSCs is clinically carried out using granulocyte colony –stimulating factor (G-CSF) along with cyclophosphamide as stimulants. However, following mobilization HSCs home back to the bone marrow indicating stem cell release and the subsequent homing is a sequential process playing a vital part in animal/human physiology.

HSCs present within the bone marrow constantly produce high levels of lymphoid and myeloid blood cells (with limited lifespan) which are released into the circulating blood while stem cells maintain their undifferentiated state. But a closer look suggests that a very small amount of quiescent progenitor cells are also released into the peripheral bloodstream. Out of the multiple theories suggested for HSC mobilization, one suggests that mobilization enables the constant repopulation of progenitor cells within the constantly changing bone ultra – structure (i.e. bone degradation & formation). Clinical or experimental mobilization of stem cells can be induced by cytokines such as G – CSF, GM – CSF, interleukin (IL) – 7, IL – 3, IL – 12, stem cell factor (SCF) and chemokines such as IL – 8, Mip - 1α, Groβ and chemotherapeutic agents like cyclophosphamide and AMD1300 (Plerixafor). Stem cell mobilization followed by CD34^+^ isolation has become a major source of stem cell transplantation [12].

HSC mobilization and homing are both regulated by the internetworking of cytokines, chemokines, and proteases. Mobilization of HSCs is mainly brought about by the loss of cell to cell contact (due to the downregulation of cell adhesion molecules) and desensitization of chemokines signaling, mainly the SDF – 1/ CXCR4 axis. On the contrary, upregulation of cell adhesion molecules and activation of the chemokines signaling pathway (SDF – 1 / CXCR4 axis) is responsible for HSC/stem cell homing. Lastly, it may be said that a better understanding of all the involved signaling cascades is required for a better understanding of stem cell mobilization and homing [13].

### Stem cell differentiation and plasticity

Classification of stem cells based on their differentiation potential has been mentioned earlier in this review [1]. With regards to stem cell plasticity it may be mentioned that initially it was believed that stem cells housed in a particular tissue could only differentiate into specific cell lines of that particular tissue type. For e.g. neural stem cells would only generate neural cells. Recent studies and experimental evidences have proven that embryonic and adult stem cells are more plastic than previously considered. E.g. Irradiated mice when injected with neural stem cells have shown reconstitution of hematopoiesis. Such examples have also been seen in drosophila when cells were transplanted between the imaginal discs (undifferentiated cells that form legs and wings in drosophila) some transplanted cells acquired the positional identity of the new location [14] and also, in humans where XY liver cells were seen in women receiving male hematopoietic stem cell transplants, suggesting hepatocyte generation from HSCs [15, 16]. Reasons implicated for such plasticity is transdifferentiation, where a differentiated cell takes on another differentiated phenotype or alternatively, stem cells first differentiates into a common progenitor cell before redifferentiating into another distinct cell types. In conclusion it may be said that stem cells establish and maintain their differentiated state via epigenetic signals. Changes in their lineage are brought about not only by nuclear transfer or cell fusion but their immediate milieu and extracellular signals [17].

### Neural stem cells as a model for stem cell development

It has been seen in mouse development that the spinal neural tube at day 8 (E8) of embryonic development consists of over 50% stem cells and at day 10 (E10) of embryonic development the telencephalon contains 5 – 20% stem cells. However, with further embryonic development these stem cells start yielding progenitor and differentiated cells as a result the stem cell pool is diluted. E.g. there is a 40% drop in stem cell concentration in the spinal neural tube at E12 and at postnatal day 1 the stem cell concentration drops to 1%.

During development the body axis pattering occurs due to signaling systems that impart positional information. Thus, it may be said that signaling molecules can control the regional specificity of progenitor cell populations if progenitor cells respond differently to different concentrations of signals. In case of the nervous system the prominent pattering in anterior – posterior and dorsal – ventral axes occurs early accompanied with neural induction. In vertebrates the study if neurospheres isolated from different regions of the CNS show region specific markers thereby indicating regional specificity of stem cells in early development. Similarly, basal forebrain stem cells when cultured show formation of neurons expressing high concentration GABA (γ-amino butyric acid). Hence, vertebrate stem cells seem to be positionally specified.

Apart from positional information stem cells are also guided by temporal information which is seen progenitor in progenitor cells during stage developmental changes, e.g. mid or hind brain progenitors are unable to differentiate in telencepahalonic progenitors after E13.5 in mouse. As developmental stages proceed the neural crest stem cells produce fewer neurons as compared to early stage neural plate, additionally, the range of neurons generated by the late neural crest is also restricted.

With regards to signaling it has been seen in mammals that during different stages of development stem cells react differently to signaling molecules. Signaling molecules like FGF, BMP and Noggin have been implicated to influence neural stem cells from neural induction through adulthood, but stem cell response to these factors vary with stages. A similar example is seen in drosophila where transcription factors such as hunchback, kruppel, castor and grainyhead regulates production of different neurons at different times. Such modeof action could possibly be controlled by a cell intrinsic timing mechanism.

Perhaps, just like in the nervous system stem cell development in general is influenced by the accompaniment of environmental cues along with intrinsic timing mechanism [18].

### How stem cells may affect other cells by releasing microvescicles

The use of stem cells in the treatment of various diseases and injuries has gained much prominence in the past few years. Injected stem cells like mesenchymal stem cells (MSCs) have been implicated in repairing injuries and stimulate tissue regeneration through the secretion of soluble factors that regulate various processes of tissue regeneration, like host cell proliferation, cellular apoptosis, inflammatory responses, angiogenesis etc. Recently it has been brought to light that stem cells use membranous small vesicles, collectively called microvesicles to repair damaged tissues. It has been long established that microvesicles are secreted by many types of cells and exist in almost all types of body fluids. These microvesicles serve as vehicles to transfer protein, messenger RNA, and micro RNA to distant cells altering gene expression thereby regulating cellular proliferation and differentiation of the recipient cells. *In*-*vitro* studies and studies on animal models have suggested promising application for microvesicle mediated regenerative therapy, its efficacy and feasibility in clinical medicine is yet to be established. Further studies on the subject could possibly lead to novel approaches in regenerative medicine.

Until recently the study of cell-cell communication has been confined primarily to the context of chemical and physical signals in the form of soluble proteins, bioactive lipids, gases, and electrical impulses. More recently, evidences have been cited to show that membranous vesicles released from multiple cell types also contribute to cell to cell communication. Exosomes and microparticles are two such populations of membranous vesicles released from cells.

#### Exosomes

Exosomes are small (50–100 nm) membranous vesicles that arise in the endocytic pathway and are released by numerous cell types, including neurons, immune cells, cancer cells, and stem cells. As soon as Exosomes mature they become multivesicular bodies (MVBs) by accumulating intraluminal vesicles through the invagination of the limiting membrane of the endosome. MVBs destined for degradation fuse with lysosomes, but a subset of MVBs fuse with the plasma membrane and release intraluminal vesicles into the extracellular environment as exosomes. Exosomes are enriched in heat shock proteins (Hsp70 and Hsp90) as well as endosome-specific proteins, such as Alix and TSG101. In addition, exosomes contain cholesterol, ceramide, integrins, and tetraspanins (CD9, CD63, and CD81), all of which are typical components of microdomains in the plasma membrane, called lipid rafts. Lipid rafts are rigid membrane domains involved in sorting lipids and proteins during endocytosis.

#### Microparticles

Microparticles (100–1000 nm) are shed vesicles that arise from the budding of the plasma membrane through the dynamic redistribution of phospholipids. Plasma membrane reorganization and microparticle budding are triggered by increased intracellular Ca21 concentrations and subsequent activation of several key enzymes— notably, calpain, scramblase, and floppase. Microparticle secretion has been best characterized in platelets, red blood cells, and endothelial cells. Microparticles lack proteins of the endocytic pathway but are enriched in cholesterol and lipid raft-associated proteins, such as integrins and flotillins. Although tetraspanins are used commonly as unique markers for exosomes, they can be detected in microparticles in some cases. This overlap in molecular markers makes the distinction between exosomes and microparticles sometimes ambiguous and, therefore, both types of membranous vesicles is collectively be referred to as microvesicles (MVs).

MVs have been isolated from many types of biologic fluids, including serum, cerebrospinal fluid, urine and cancer cells by use of ultracentrifugation and size exclusion column chromatography. The physiological functions of MVs in many tissues remain largely unknown; however, their potential roles in pathologic setting have been widely studied in oncology and immunology. A subset of molecules, including proteins and RNAs, have been identified in association with MVs, and their effects on recipient cells have been studied intensively in these fields, primarily in vitro.

#### MSCs and regenerative medicine

Although multiple types of stem cells have been established for use in regenerative medicine (embryonic stem cells, MSCs, and induced pluripotent stem cells), the most commonly used type of stem cells in regenerative medicine is autologous MSCs due to fewer technological and ethical constraints. Recent findings also indicate a proregenerative role for MVs released by MSCs (MSC-MVs) in several models of tissue regeneration, including regeneration of kidney, heart, liver, and nervous tissues.

#### MVs and ESCs

Because of their potential use in medical applications, ESCs have been studied extensively during the past decade, although MVs released from ESCs (ESCMVs) have not attracted wide interest. ESC-MVs contain mRNA encoding Oct4, SOX2, and Rex1, which are key genes for pluripotency, as well as the Oct4protein. After incorporation of ESC-MVs, hematopoietic progenitor cells translate mRNA encoding Oct4, the master transcription factor for pluripotency, although this has not been shown to result in pluripotency of the recipient cells.

#### Future perspective

The functional significance of MVs in regenerative medicine has yet to be firmly established. Although many studies have demonstrated that MVs isolated from various cells are capable of supporting tissue regeneration, it is not clear whether MVs are indeed necessary components of the regenerative process. To prove this, release or incorporation of MVs needs to be blocked specifically in vivo, which requires a deeper understanding of their synthetic pathways, selective uptake of their contents, secretion, and incorporation by target cells. Fortunately, possible molecular targets involved in MV biogenesis and secretion have been elucidated recently in vitro [19].

### Model of stem cell research

#### Drosophila melanogaster

Earlier in this review it has been mentioned that the formation and maintenance of stem cells depends on its surrounding support cells along with extracellular secretions. However, studies in drosophila have shown new niches that lack a stable population of support cells [20].

#### Drosophila germ line stem cells (model of niche regulation)

Germ line stem cells (GSCs) are retained by both drosophila sexes during most of their adult life. Although, drosophila ovary and testis have different organizations the arrangements of their respective GSC niches share architectural similarities. In case of the ovarian niche 2 – 3 female GSCs (fGSCs) are in constant contact stromal hub cells and in case of testis approximately 8 male GSCs (mGSCs) are in contact with stromal hub cells. Both cap and hub cells are implicated in forming the respective stem cell niches. Pearson *et al*. have stated that in drosophila, stromal stem cells form a stem cell independent microenvironment mainly because of their organization, cell – adhesion properties and extracellular – signal expressions. Thus, cells that are capable of responding the niche environment because of their intrinsic mechanism are able to populate and self renew. This phenomenon has been seen in both male and female drosophila GSC niches which includes the actual GSCs and the differentiating germ cells. This model has enabled researchers to outline the niche functionality in several species where stem cells are maintained in niches with similar properties [20].

#### Drosophila somatic stem cell niche (intestine and ovaries)

Intestinal stem cells (ISCs) in drosophila midgut have been determined using genetic lineage markers. The ISCs associated with the basement membrane along with their daughter cells via armadillo rich junctions. However, ISCs don’t seem to be connected to any stromal cell types suggesting existence of self renewing stem cells which are not characterized by stromal cells. It may also be noted that the differentiation of daughter cells depends on Notch signaling as is seen in neural stem cells suggesting intrinsic factors at play in ISC stem cell renewal.

In case of the ovary, existence of stem cells in the ovary has been known for long. The ovary has been stated to have two types of somatic stem cells namely, Follicle stem cells (FSCs) and Escort stem cells (ESCs). FSCs are present in the germarium (2 FSCs/germarium) that encapsulate the 16 – cell germ cyst and plays a major role in determining the polarity of the developing oocyte. It was recently found [21] that FSCs like ISCs, lack stable stromal cell contact and FSC daughter cells displace other FSCs within the same germarium indicating that the microenvironment of each FSC might be a niche and the intrinsic factors expressed by the FSCs regulate their asymmetric division and extracellular environment. In further studies [22] it has been stated that a number of signaling pathways may be involved in controlling FSC self – renewal and maintenance, however these signaling molecules are produced by distant cells which are also associated with regulating the fGSC niche, suggesting that specialized support cells don’t necessarily have to be in contact with their target stem cells to regulate the nice and its activity [20].

#### Drosophila neuroblasts

Drosophila neural stem cells also called neuroblasts (NBs) have been implicated as stem cells without a niche. The NBs right from embryogenic stages through to larval stages gives rise to an array of sensory tissues. Approximately 60 NBs divide to form 2 daughter cells, one of which is the larger, apical daughter which remains as NBs while the basal cell transforms into the ganglion mother cell (GMC) which then undergoes further division prior to differentiation.

NBs also have a strong association with the epithelial cells in order to maintain their proper polarity and (or) cell division with regards to their neighbouring cells suggesting, extrinsic cues have a hand in NB division. However, studies by Yu *et al*. [16] have shown that in comparison to GSCs NB self renewal and GMC production is regulated by intrinsic factors involving polarity, the mitotic apparatus and distribution of fate determinants. Thus, suggesting NB self renewal and regulation is independent of the niche [20].

#### Drosophila hematopoietic stem cell niche

Recent studies and publications have identified hematopoietic precursor (HP) cells in the embryonic and early larval stages which form haemolymph cells. The evolution of molecular markers has enabled scientists to identify and locate regions of the drosophila lymph gland where hematopoiesis occurs and is controlled by a group of cells known as the posterior signaling centre (PSC). Similar to the ovarian GSC niche the contact between PSCs and HP cells seems to be the most important factor in the maintenance of stem cells thereby suggesting that the probability of PSCs forming the stromal component of the niche [20].

#### Multipotent stem cells in drosophila kidney

The drosophila renal organs also known as malpighian tubules (MT) seem to contain proliferating stem cells in the proximal segment. Scientists using lineage tracking technique have managed to identify a small subpopulation of “small nuclear” cells in the proximal segment which are multipotent and are termed renal and nephric stem cells (RNSCs). These cells have been implicated in differentiating into renalcysts in the proximal segment and type – 1 and 2 cells in the upper tubule segment. Also, strong JAK – STAT signaling plays a major role in specifying MT cell lineage while the weaker JAK – STAT signal plays a role in the formation of RNSC daughter cells (renalblasts).

RNSCs therefore don’t seem to have an organized niche system or associate with any cell type, rather, RNSC self – renewal seems to be regulated by JAK – STAT signaling. Nevertheless, scientists are studing this model further to determine if there are any intrinsic or other unidentified extrinsic cues which influence RNSC differentiation and self – renewal [20].

Apart from niche related studies, other studies in drosophila have shown stem cells to actively participate in maintain the niche environment. This has been shown by the requirement of constant Notch signaling to from FCS to maintain stromal stem cells. In absence of this signaling cascade the stromal stem cells seem to disappear. Also, cell adhesion and the extra cellular matrix (ECM) also play a major role in niche morphogenesis monitoring stem cell migration, rearrangement and formation of structures e.g. the epithelial sheet. *D*E-cadherin, β- catenin and GTpase to some extent play an important role in this regard [20].

In conclusion it may be said that drosophila and drosophila stem cells act as a simple model which can be extrapolated to understand more complex animal or human models and therefore, drosophila has immense significant value in the study of stem cells, their niche and their mode of function.

#### Concluding remarks

Individuals suffering for lifelong threatening conditions are hoping for some cure or amelioration of symptoms through the use of stem cells as therapy. It is therefore, the need of the hour for both scientists and physicians to understand stem cells in a way that will enable them to optimize the use of stem cells in treating complex ailments [1].

## Perspective 2. Stem cells in higher organisms and stem cell niche in mouse

Stem cells are a subset of cells that have the unique ability to replenish themselves through self-renewal and the potential to differentiate into different types of mature cells. These characteristics thus play a major role in tissue generation during embryogenesis and during tissue damage. There are two main types of stem cells: embryonic and adult. The pluripotent embryonic stem cell is derived from the inner cell mass of blastocysts and has the ability to give rise to all the germ layers- endoderm, mesoderm and ectoderm. As embryogenesis progresses, the need for organogenesis arises and the embryo forms germ line stem cells for reproduction and somatic stem cells for organogenesis.

### Terminology of stem cell niche

After birth, adult stem cells, including both GSCs and SSCs, reside in a special microenvironment termed the “niche,” which varies in nature and location depending on tissue type. The word ‘niche’ may refer to a recess, and in ecology it refers to a place where an organism can reside and reproduce. In case of stem cells, niche may refer to a place of dwelling of the cells where they can be awakened by some stimulus. But the simple location of stem cells is not sufficient to define a niche, the niche must have both anatomic and functional dimensions, specifically enabling stem cells to reproduce or renew. Several factors are important to regulate stem cell characteristics within the niche: cell-cell interaction between stem cells, as well as interaction between stem cells and neighboring differentiated cells, interaction between stem cells and adhering molecules, extracellular matrix components, oxidative stress, growth factors, cytokines and physiochemical nature of the environment including pH, ionic strength and metabolites, like ATP are also important. The stem cell and niche may induce each other during development and reciprocally signal to maintain each other during adulthood.

### First hypothesis and evidence of stem cell niche

In 1978, Schofield proposed the “niche” hypothesis to describe the physiologically limited microenvironment that supports stem cells. The niche hypothesis has been supported by various types of coculture experiments in vitro and by bone marrow transplantation. However these studies did not resolve the issue of the exact stem cell location and niche structure in vivo.

Although locating stem cell niches in mammals had been difficult because of the extreme complex anatomic structures, other genetic models (Drosophila and C. elegans) have been fruitful in studying stem cells and their locations. In 2000, the germinal tip adjacent to GSCs was defined as the niche supporting GSCs in Drosophila ovary, whereas the hub located at the tip of the drosophila testis served this function in testes.

First in mammals, the epithelial stem cell location was successfully identified in the bulge area of hair follicle, and the intestinal stem cell location was found near the crypt base. These were identified by the stem cells ability to retain BrdU and radioactive thymidine labels.

Recently, there has been significant progress in knowledge regarding stem cells and their surrounding microenvironment. These were known from a variety of mammalian models. Two independent, simultaneous studies using genetic mutant mouse models led to the identification of osteoblastic cells as the key component of HSC cells.

### Role of extracellular matrix in regulating stem cell niche

The long standing concept of extracellular matrix regulating stem cells can be verified by a few examples in mammalian stem cell system. For example, in the skin where the beta-1 integrins are known to be differentially expressed on primitive cells and to participate in constrained localization of a stem-cell population through presumed interaction with matrix glycoprotein ligands.

### Stem cell niche of lungs and expression of stem cells of lungs following mechanical injury

#### Introduction

The field of stem cell biology continues to grow as numerous types of stem cells are identified in animal models and in human tissues. Clearly, the identification of these previously elusive cells has led to models redefining the development of issues and lineage relationships that exist between adult cells [23–25]. The development of Fluorescent associated cell sorting (FACS) has been crucial to the isolation of rare stem cell population from adult tissues. Methods to disaggregate complex tissues and grow these tissues in culture and the ability to perform molecular analysis on small number of cells have also allowed the stem cell to expand.

The identification of stem cells relies on their definition. Tissue stem cells are unspecialized cells that are capable of cell renewal and give rise to specialized or differentiated cells. In some tissues, cells exhibiting stem cell characteristic has been identified during a particular developmental stage or in the adult tissue after injury. Importantly for most tissues, a direct lineage relationship between putative embryonic and adult stem cells has never been established. Many stem cells maintain proliferative capacity for long durations of life of an organism yet are quiescent in normal tissue, and only a fraction of stem cell population may enter cell cycle after injury. The most distinguishing feature of stem cell is self-renewal.

Some organs such as hair follicles, blood and gut, which constantly renew themselves throughout life, contain adult stem cell that are morphologically unspecialized, have a relatively low rate of division and are topologically restricted to regions known as ‘niches’ that are tightly regulate their behavious [24, 26, 27]. These dedicated stem cells undergo long term self renewal.

In contrast to rapidly renewing organs such as the skin and gut, some organs apparently maintain themselves without the aid of an undifferentiated stem cell population. Evidence for this concept comes from recent experiments in which insuling-producing beta-cells of the adult mouse pancreas were labeled with a heritable genetic marker and followed during normal turnover and regeneration after partial pancreatectomy [28, 29]. Likewise, in the liver, turnover and regeneration after hepatectomyinvolves the division of differentiated hepatocytes. However, if hepatocyte proliferation is inhibited, interlobular bile duct cells can replenish the hepatocyte population. Such observations have endangered the concept of facultative stem cells- normally quiescent differentiated cells can act as stem cells after injury, perhaps by recapitulating processes that are active during development.

The adult lung is a vital and complex organ that normally turns over very slowly. The epithelial cells that line the airways are constantly exposed to potential toxic agents and pathogens in the environment, and they must therefore be able to respond quickly and effectively to both cellular damage and to the local production of immune cytokines. Over the years, several experimental protocols have been developed in mice that mimic the injuries and rapid repair processes elicited in the lung by environmental challenges. The picture that is emerging from these models is that different regions of the respiratory system-the trachea and large airways, and the distal bronchioles and alveoli harbor and use different kinds of stem cells and strategies for maintenance and repair. Moreover, there is evidence that differentiated epithelial cell types are able to proliferate and transdifferentiate in response to some conditions. However the precise mechanisms involved in any of the processes are still very unclear.

#### Evidence of lung stem cells

The pulmonary system contains a variety of epithelial cell populations [2]. In human, basal cells, secretory goblet cells, submucosal glands, and ciliated cells line the trachea and upper airways. The same regions in the mouse are populated by ciliated and nonciliated columnar cells, and a few submucosal glands are found in the proximal airway. The murine submucosal glands includes mucous-producing cells, ciliated cells and basal cells. Neuroendocrine cells are found mostly within the large proximal airway and reside in clusters refrred to as neuroendocrine bodies. The non-ciliated, columnar Clara cells that line the bronchioles and terminal bronchioles secrete surfactants to aid in oxygen exchange and provide a protective epithelial barrier in the airways. The alveolar epithelium is composed of alveolar type-II cells (AT2), the cuboidal epithelial cells that produce surfactants and the resulting surface tension required for gas exchange, as well as the alveolar type II (AT1) cells, the flat epithelial cells that deliver oxygen to the blood. Numerous stromal cells are present, and the lung has been described as containing at least 40 different cell types.

#### Evidence from ex vivo studies

In the hematopoietic system, it is possible to test to test the ability of cells to restore all the blood cell lineages by injecting them intravenously into an irradiated host. Likewise, dissociated hepatocytes can repopulate the damaged liver after injection into the portal vein, and clonal analysis can be achieved in the system using retrovirally labeled cells. Recent studies show that a complete mouse mammary gland can be made from a single adult epithelial cell implanted into a mammary fat pad [30, 31]. Two relatively older methods (ex vivo) have been used to examine the regenerative potential of isolated lung epithelial cells: the rat tracheal xenograft model and cell culture. These systems are particularly useful because they can be applied to the study of human adult and fetal airway epithelial cells, including tracheal cells and nasal polyps.

#### Rat tracheal xenograft model

In this model , onto the surface of host rat trachea that has been denuded of endogenous epithelial cells by freeze-thawing, epithelial cells isolated from donor airway epithelium are seeded after dissociation. The trachea is then grafted subcutaneously into an immunodeficient mouse. Several weeks later, the restoration of a well –differentiated, normal airway epithelium with a few submucosal gland can be observed, though it is not known whether this organization can be maintained over long term.

The second experimental approach has been to sort the donor cells into basal and non-basal populations and then to follow their ability to reconstitute the surface to reconstitute the surface epithelium. The results from these studies have been so far very variable. Some suggest that both population can restore tracheal epithelium equally well [32, 33]. However, others have found that only columnar or only basal cells can restore all the epithelial cell types. These discrepancies may be due to differences in sorting methods, donor species or the length of time allowed for epithelial repopulation. In spite of these differences, these results suggest that both the columnar and basal cells can restore the tracheal epithelium in the xenograft model.

These xenograft studies are limited as the host tracheal mesoderm is dead and may not provide an ideal environment for the survival and differentiation of all donor lung cells. A potential alternative, which has been more recently explored, is to place dissociated adult lung epithelial cells together with embryonic lung mesenchyme and the graft the recombinant under the kidney capsule of immunocompromised mouse. In this environment, the graft are readily vascularized. There is a close association between lung cell precursors and blood vessels, which is crucial for the normal development, has been established in recent studies.

#### Injury by naphthalene

A popular lung injury model is the destruction of Clara cells by naphthalene. This is an aromatic hydrocarbon, which is usually intraperitoneaally injected, from where it reaches the lungs with the help of flowing blood. Napthalene kills only the cells that express cytochrome p4502F2, which is converted to toxic epoxides in such cells. All the Clara cells die within a few hours, except the cells which do not express P4502F2, and are therefore resistant. Under the dying normal Clara cells, these cells quickly spread out in an attempt to cover the basal lamina and maintain the permeability barrier of the epithelium. Cell proliferation begins 2–3 days after the injury, and by 2–4 days the epithelium has returned to steady state [34, 35]. Different mechanisms appear to operate for the renewal of the Clara cells, depending on the region where the repair occurs.

#### Response to naphthalene injury to lung

In the more distal lung, it has been studied that Clara cell population is restored by the proliferation and self-renewal of a small number of resistant or ‘variant’ (Clara v) cells after naphthalene injury. These are label-retaining subpopulations of Clara cells located adjacent to cluster of neuroendocrine bodies (NEBs) in the bronchioles [10] or at the broncho-alveolar duct junctions (BADJs), where there are very few neuroendocrinal bodies [9, 10, 36]. The lack of expression of cytochrome P450 on the Clara ‘variant’ may represent less differentiated Clara cells, when related to majority of Clara cell population. It can be stated that, if Clara ‘variant’ (and putative BASCs) can be shown to self-renew and give rise to differentiated cells, they can be classified as dedicated stem cell, rather than as facultative stem cells which responds to injury by naphthalene. The notable difference between Clara ‘variant’ or putative BASC to that of classical undifferentiated stem cells is – these cells often express markers of differentiated cell whereas the later do not.

The factors that instigate the self-renewal and lineage diversification of the Clara ‘variant’ cells are current not complete understood. A question remains whether Clara ‘variant’ (and putative BASC) are the only cells that can regenerate after naphthalene injury. To address this issue, Clara cells were killed with ganciclovir to a transgenic mouse strain expressing the herpes simplex thymidine kinase specifically in Clara cells [10, 37, 38]. After this airways could not be repaired. This results suggests that ciliated cells cannot give rise to Clara cells. However, other claims has been made that flattened ciliated cells can proliferate 48 hrs after naphthalene injury. They also suggested that ciliated cells can transdifferentiate to non-ciliated columnar cells. That is, it can be suggested according to the last authors that, after injury, ciliated-cells can both self renew and give rise to cells of other lineage, i.e., they behave as classical stem cells. If ciliated cells do not normally self-renew and are quiescent, but does so under these conditions, to be facultative stem cells.

#### Tracheal injury model by inhaled Sulphur dioxide

Only subsets of epithelial cells in the lung are injured by both naphthalene and oxidant exposure. Ventura et al., [39] have identified specific niches of stem cell expansion that are marked by distinct zonal boundaries. They used sulfur dioxide inhalation in mice to destroy the majority of the pseudostratified epithelial cells in the upper trachea, leaving protected cells behind in the surface layer and submucosal glands, and in pathches of denuded basement membrane. It was observed within 7 days that, full repair has taken place and a morphologically normal epithelium is re established. In order to identify the dedicated stem cells in this model, it was assumed that such cells divide less frequently and will retain a DNA label aver a long time period. So experimental animals were subjected to repeated rounds of sulfur dioxide and BrdU, so that almost every epithelial cell become labeled. After a period of about 3.5 months small groups of label retaining cells were located in the collecting ducts of submucosal glands or to the surface epithelium in the inter-cartilage regions. It was observed that morphologically the label retaining cells in most regions were basal cells. These experiments were supported by the naphthalene recovery system discussed earlier. Moreover, these cells can repopulate the entire tracheal surface of a xenograft model in which the epithelium was completely denuded [40].

#### Repair after damage by inhaled oxidants

Although naphthalene injury was used to determine the regeneration of lung cells from clara ‘variant’ cells, other models specifically destroy ciliated cells or type I alveolar cells. This includes inhalation of oxidants such as nitric oxide and ozone (which selectively kills ciliated cells), and administration of the chemotherapy agent bleomycin (which kills type I cells). These studies can assert that ciliated cells can be regenerated from clara cells, and AE I type cells can be regenerated from AE II cells.

#### Lung stem cell niche: potential

From the studies as described above, it can be asserted that the cells which retain labels, appear to reside in the intercartilage region, where there are abundance of blood vessel and nerves. It has been suggested that these non-epithelial cells are a part of the special ‘tracheal niche’ [40] and it regulates the activity of dedicated stem cells. Also the assertion that broncho-alveolar duct junction can act as a niche for putative BASC comes from the work of Giangreco et al., and Reynolds et al. These regions are also supplied with abundant blood vessels. These ideas can certainly be related to other systems like hair follicle, intestine, bone marrow and brain, where much more molecular evidence for regulatory signaling between stem cells and surrounding differentiated cells has been found [41–43]. The genes active in hair follicle stem cells are Wnt, Bmp, Fgf intercellular signaling pathways [44, 45]. These are also associated with signaling during lung development and can be likely components of the stem cell niche of lungs and trachea.

#### Scope of further research

The control of the stem cell behavior by a niche, as has been shown in recent studies can be of immense importance in the field of developmental biology. Developmentally evidence of endothelial cells regulating the proliferation and differentiation of organ primordial are clear, and also organ morphogenesis (kidney) [46]. Likewise, much recent studies involving damaged lung stem cell regeneration has been performed using hESC derived cells, which may involve the regulatory and self renewing role of BASCs [47, 48]; Studies in one system can promote understanding in the other.

Many challenges still remain, like, the identification of more phenotypic markers for lung cells to allow unambiguous identification and efficient sorting by flow cytometry. There may be many more important subpopulation of epithelial cells that may have been completely missed. Also needed are techniques for identification of development potential of isolated lung epithelial cells. Also the specific interaction and the mechanism involved between epithelial and non epithelial cells during tissue damage and repair, and homeostasis must be better understood. These answers will most likely come from different directions. Further researches in developmental biology will most probably show the path.

#### The epithelial/hair follicle stem cell niche

A well organized architecture can be found in skin, with its appendix hair follicle structure. This provides an excellent system for studying the molecular mechanisms that regulate stem cell renewal, proliferation migration and lineage commitment. Each hair follicle is composed of a permanent portion, including sebaceous glands and the underlying bulge area, and a dynamic renewing portion, which undergoes cycles of a period of active growth (anagen period), apoptosis driven retraction (catagen phase) and a short period of rest (telogen period). The bulge area functions as a niche, where epithelial stem cells are located and maintained. These stem cells are multipotent and give rise to daughter cells that either migrate upward to serve as epidermal progenitors for generating epidermal cells during wound repair or migrate downward to convert to hair-matrix progenitor, which further give rise to the hair shaft.

During the early anagen phase, the dermal papilla region may provide the dynamic signals that activate stem cells; however, the cellular components of the niche in the bulge are yet to be defined other than as stem cells per se. The dermal sheath derived from mesenchymal cells adjacent to the epithelial stem cells in the bulge area most likely provides the niche formation.

#### Potential of cellular regeneration from hair follicle stem cells

Adult mammalian stem cells were previously thought to differentiate exclusively into cells of their tissue of origin. A number of recent reports have shown that tissue based adult stem cell therapy is more flexible than previously thought. Although most of the previous hair stem cell functional studies were performed using rodent tissue, later studies have developed methods to isolate human adult stem cells from human hair follicles in a human embryonic stem cell culture condition. It has been shown that isolated cells are capable of differentiating into neurons, smooth muscle cells, and melanocytes in specific induction medium. Those cells not only express lineage specific markers but also show appropriate functions in ex-vivo conditions. These cells appear to be located in the bulge area of human hair follicles.

#### Isolation of hair follicle stem cells: Procedure

Human hair follicles can be isolated from human subjects, both dead and alive. The tissues can be processed (rinsed, trimmed to remove excess adipose tissues, cut into small pieces, and subjected to enzymatic dissociation in dispase containing medium), and after that, the hair follicles can be plucked from the dermis. After rinsing with PBS, the follicles can be examined under microscope. To obtain viable single cells from follicular epithelium, hair follicles can be grown in human ESC media containing 80% knockout-DMEM, 20% knockout serum replacer, 220 mmol/lit L-glutamine, 0.1 mmol/Lit BME, and 4 ng/ml basic fibroblast growth factor. This particular media is to be mEF-conditioned, by growing mouse embryonic fibroblast in the media prior to growing hair follicle in it.

After growing the follicle cells in hESC media in 96 well plates, from each wells containing more than one cells, cells were taken and grown on mEF feeder layer. After that embryoid bodies were formed from the grown cells.

#### Expression of various stem cell markers

To characterize cells in hair spheres, gene and protein expression can be studied. In hair follicular stem cells, the expression of nestin (NES) and other genes transcribed in embryonic neural crest stem cells were analysed by real-time RT-PCR. From recent studies, it has been found that hair spheres expresses NES as well as the neural crest stem cell markers SLUG, SNAIL, TWIST, SOX9 and BMP4. Also atleast 10% of cells were found to be nestin positive by nestin immunocytochemical staining on hair spheres.

#### Differentiation of stem cells to different lineage specific cells

Stem cells obtained from hair has been found to have the potential to differentiate into several types of cells. This is possible by growing the stem cells in differentiation medium. For smooth muscle differentiation, dissociated cells were cultured in a medium containing 90% knockout DMEM, 1% nonessential amino acids solution, 10% FCS, 10 ng/ml transforming growth factor-beta 1. Melanogenic differentiation medium differentiated hESCs into melanocytic lineage exclusively. The media contains dexamethasone, insulin-transferrin-selenium, linoleic acid-bovin serum albumin, low-glucose DMEM, L-ascorbic acid, conditioned media of mouse L-Wnt3a cells, stem cell factor and bFGF.

#### Differentiation to melanocytic cells

When observed after about 2 weeks after growing in specific differentiation medium, in case of melanocytic cells about 40% of cells in medium has been found to adopt a dendritic morphology typical of melanocytes. Real-time RT-PCR can be done to reveal that the differentiated cells gain the expression of melanocyte markers MITF and TYRP1, whereas they lose expression of stemness gene NANOG. Melanin pigment can be detected in differentiated cells by Fontana- Mason staining. In order to study whether follicle stem cells derived melanocytes are have acquired normal function, differentiated melanocytes can be introduced into human skin reconstructs that mimic human skin architecture. It has been studied that, in the human skin environment, human follicle derived melanin pigment not only produce melanin pigment but also respond to skin patterning cues in ways similar to those of normal epidermal melanocytes.

#### Differentiation to neural cells

Studies show that, after about 2 weeks of growing in neural differentiation medium, about 10% of cells show long dendritic processes and express MAP2, NFM and chromogranin A proteins. The expression of MAP2 and beta3-tubulin (TUBB3) genes were detected by real-time RT-PCR. The gain in neural markers was accompanied by a loss of NANOG gene expression after neural differentiation.

#### Smooth muscle cell differentiation of human hair follicle cells

Recent studies has shown that, in smooth muscle differentiation medium, approximately 80% of hair follicle cells has been induced to smooth muscle cells that have acquired abundant cytoplasm. Different smooth muscle markers like CNN3 and DES were also detected by real-time RT-PCR, accompanied by loss of NANOG gene expression.

## Investigation of lung stem cell niche by coaxing cells out of lung niches in IPF model in mouse

### Introduction

Tissue injury and repair are ongoing processes in the lung and result from acute and chronic exposure to environmental insults. There are a myriad of effectors of lung injury, including infectious agents, particulate and chemical pollutants, radiation, and host defense mechanisms gone awry. Many of these processes are ablative in nature and require repair mechanisms that regenerate mature lung tissue through cell proliferation and differentiation.

Fundamental to understanding mechanisms of repair are identifying and characterizing the cells that are potentially capable of repopulating the injured tissue. Currently, efforts are being made to identify *1*) which cell (s) repopulates regions of injured lung; *2*) what their source is (endogenous or resident cells vs. exogenous or recruited cells); and *3*) whether they are pluripotent stem cells capable of self renewal or transient amplifying cells that are multipoint but more lineage committed. In the lung, multiple cell populations contribute to lung repair [37]. Most, like the basal cells of the tracheal epithelium, alveolar type II cells, bone marrow-derived stem cells, and residential stem cells that potentially serve the vascular compartment appear to be anatomically localized [26, 42]. Others, like the side population (SP) cells, have not yet been localized to a single lung compartment.

The molecular phenotype of hematopoietic stem cells (HSC) has been extensively characterized and is defined as a population of cells that are CD45+, Sca-1+, c-kit+, and Lin–. HSC are further characterized by their ability to rapidly efflux the DNA dye Hoechst 33342 [45, 49].

The existence of putative lung tissue stem cells has only been suggested relatively recently through the use of rodent injury models in which abundant progenitor cells are depleted through either chemical or physical means [34, 42, 45, 50]. At least three distinct regions have been described that support populations of lung tissue stem cells: intercartellagenous regions of tracheobronchial airways [42], neuroepithelial bodies (NEB) in bronchioles [47], and the bronchoalveolar duct junction (BADJ) [34, 47]. Each region harbors putative tissue stem cells that share the common properties of a relatively undifferentiated phenotype, infrequent proliferation (demonstrated through use of DNA label retention assays), and a differentiation potential that is appropriate for the compartment in which they reside. A combination of immunophenotypic and cell ablative strategies has been employed to demonstrate that bronchiolar stem cells residing within both the NEB and BADJ microenvironments represent a Clara cell secretory protein (CCSP)-expressing variant Clara cell, and that the BADJ-associated population are dual positive for both CCSP and pro–surfactant protein C. Recent studies by Kim and colleagues suggest that CCSP/SP-C dual positive cells can be enriched based upon their unique cell surface phenotype (Sca1^+^, CD34^+^, CD31^–^, CD45^–^) and maintained long-term *in vitro*[50].

The report by Summer et al. [51] adds to our understanding of the lung SP and its function by phenotypically characterizing these cells. The authors have demonstrated that there are both CD45+ and CD45– lung SP cells and that both of these populations are contributed to by the bone marrow compartment, as demonstrated by whole bone marrow transplantation experiments.

### Materials and methods

#### Mice

Both gp91^phox-/-^ mice [24] (Jackson Laboratories, Bar Harbor, ME) and MMP12^-/-^ mice were on a C57Bl/6 J background and had been outcrossed and then intercrossed for three generations to generate animals deficient in both genes. C57BL6 mice (Taconic) were used as the control group and are called wildtype (Table 1).

**Table 1:** **Total number of animals per treatment group in three independent experiments**

	Genetic knockout models		
Treatment groups	WT	NOX-/-	MMP12NOX-/-
Control(+Alum)	14	14	16
+OVA	16	15	14

gp91^phox–/–^ mice and gp91^phox–/–^ MMP12^–/–^mice were on a C57BL6 background. These were compared with normal C57BL6 mice which were called wildtype (WT). The two treatment groups were “control” (sham treated) and “treated” (OVA treated).

#### Mouse model of bleomycin-induced pulmonary fibrosis

A single intra-tracheal dose of 0.075 U/ml of bleomycin in 40 μl saline was administered (d 0), and mice were sacrificed 14 and 21d later. Mice C57Bl/6. were kept under ABSL-2 conditions approved by the IACUC of the University of Washington and monitored daily. They were housed under specific pathogen free condition and were given food and water *ad libitum*. They were sacrificed on d 14. 1 week after bleomycin adninistration, mice developed marked interstitial and alveolar fibrosis, detected in lung sections by Masson’s trichrome stain. Analysis of cell populations by enzymatic digestion by collagenase Type IV followed by cell counting in Coulter counter and subsets identified and quantified by FCM and total and differential count of H & E stained cytospin smears of single cell suspensions show loss of type II and type I alveolar epithelial cells and influx of macrophages. AEI and II were isolated following standard protocol.

#### BrdU pulse chase

BrdU is a DNA analogue. Slow cycling cells are assumed to be stem cells and pulsing of control vs. bleomycin (single i.t. dose of 0.075 U/ml bleomycin) treated WT C57Bl/6 mice over 2, 4 and 6 days i.p. at 12 hrs interval and chase over 10 weeks is expected to yield BrdU positive cells and negative cells. While negative cells are assumed to be mature regularly cycling cells, BrdU + cells after 10 weeks of chase are most likely slow cycling stem cells that started cycling late and hence retain the label latest (Label retaining cells or LRC). C57Bl/6 mice were intratracheally instilled with 0.075 U/ml bleomycin in 40 μl volume under brief isofluorane anesthesia and animals were maintained under SPF conditions in the UW animal facilities and sacrificed periodically to assess the above. The abbreviations used are: i.p. intra-peritoneal; i.t. intra-tracheal; BAL, bronchoalveolar lavage; PB, peripheral blood. BrdU is a DNA analogue. Slow cycling cells are assumed to be stem cells and pulsing of control vs. bleomycin (single i.t. dose of 0.075 U/ml bleomycin) treated WT C57Bl/6 mice over 2, 4 and 6 days i.p. at 12 hrs interval and chase over 10 weeks is expected to yield BrdU positive cells and negative cells. While negative cells are assumed to be mature regularly cycling cells, BrdU + cells after 10 weeks of chase are most likely slow cycling stem cells that started cycling late and hence retain the label latest (Label retaining cells or LRC).

#### BALf

After pulmonary function testing, the mouse underwent exsanguination by intra-orbital arterial bleeding and then BAL (0.4 ml three times) of both lungs. Total BAL fluid cells were counted from a 50 μl aliquot and the remaining fluid was centifuged at 200 *g* for 10 min at 4°C and the supernatants stored at -70°C for assay of BAL cytokines later. The cell pellets were resuspended in FCS and smears were made on glass slides. The cells, after air drying, were stained with Wright-Giemsa (Biochemical Sciences Inc, Swedesboro, NJ) and their differential count was taken under a light microscope at 40× magnification. Cell number refers to that obtained from lavage of both lungs/mouse.

#### Lung parenchyma

Lung mincing and digestion was performed after lavage as described previously (Labarge S et al.) with 100 u/ml collagenase for 1 hr at 37°C, and filtered through a 60# sieve (Sigma). All numbers mentioned in this paper refer to cell sobtained from one lung/mouse.

#### Lung histology

Lungs of other animals of same group were were fixed in 4% paraformaldehyde overnight at 4°C. The tissues were embedded in paraffin and cut into 5 μm Sections. A minimum of 15 fields were examined by light microscopy. The intensity of cellular infiltration around pulmonary blood vessels was assessed by Hematoxylin and Eosin staining. Airway mucus was identified by staining with Alcian blue and Periodic Acid Schiff staining as described previously.

#### Fluorescin-activated cell sorter (FACS) analysis

Cells from hemolysed peripheral blood (PB), bone marrow (BM), bronchoalveolar lavage (BAL), lung parenchyma (LP), spleen, mesenteric lymph nodes (MLN), cervical lymph nodes (CLN), axillary lymph nodes (LNX) and inguinal lymph nodes (LNI) were analyzed on a FACSCalibur (BD Immunocytometry Systems, San Jose, CA) by using the CELLQuest program. Staining was performed by using antibodies conjugated to fluorescin isothiocyanate (FITC), phycoerythrin (PE), allophucocyanin (APC), Peridinin Chlorophyll Protein (Per CP-Cy5.5) and Cy-chrome (PE-Cy5 and PE-Cy7). The following BD pharmingen (San Diego, CA) antibodies were used for cell surface staining: APC-conjugated CD45 (30 F-11), FITC-conjugated CD3(145-2C11), PE-Cy5 conjugated CD4 (RM4-5), PE-conjugated CD45RC (DNL-1.9), APC-conjugated CD8 (53–6.7), PE-Cy5 conjugated B220 (RA3-6B2), FITC-conjugated IgM, PE-conjugated CD19 (ID3), PE-conjugated CD21(7G6), FITC-conjugated CD23 (B3B4), APC-conjugated GR-1 (RB6-8C5), and PE-conjugated Mac1 (M1/70). PE-Cy5 conjugated F4/80 (Cl:A3-1(F4/80)) was obtained from Serotec Ltd., Oxford, UK. PE-conjugated anti-α4 integrin (PS2) and anti-VCAM-1 (M/K-2) was from Southern Biotechnology, Birmingham, Ala. Irrelevant isotype-matched antibodies were used as controls.

#### CFU-c assay

To quantitate committed progenitors of all lineages, CFU-C assays were performed using methylcellulose semisolid media (Stemgenix, Amherst, N.Y.) supplemented with an additional 50 ng of stem cell factor (Peprotech, Rocky Hill, N.J.) per ml. Next, 50,000 cells from bone marrow, 500,000 cells from spleen, 0.01 million cells from lung and BAL, and 10 μl peripheral blood were plated on duplicate 35-mm culture dishes and then incubated at 37°C in a 5% CO_2_-95% air mixture in a humidified chamber for 7 days. Colonies generated by that time were counted by using a dissecting microscope, and all colony types (i.e., burst forming units-erythroid [BFU-e], CFU-granulocyte-macrophage [CFU-GM], and CFU-mixed [CFU-GEMM]) were pooled and reported as total CFU-C. Total CFU-c per organ was calculated by extrapolating CFU-c against number of plated cells to the total number of cells in the organ.

#### Statistical analysis

Statistical differences among samples were tested by Student *t* test. *P* value less than 0.05 was considered statistically significant.

### Results

#### Bleomycin-induced Idiopathic Pulmonary Fibrosis model

**Figure 1:**
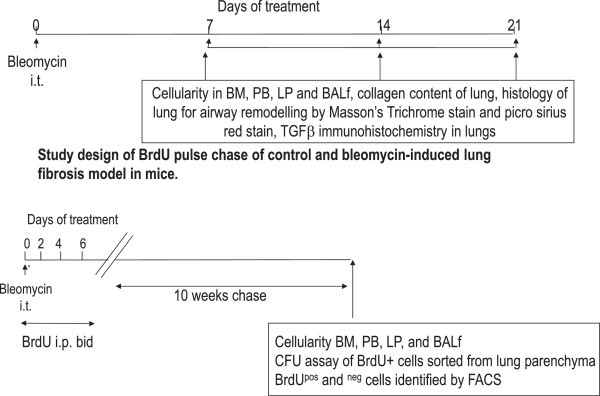
**Study design to generate fibrosis in mouse lung** **: C57Bl/6 female mice (8–10 weeks of age) were intra-tracheally instilled with 0.075 U/ml bleomycin in 40 μl volume under brief isofluorane anesthesia and animals were maintained under SPF conditions in the UW animal facilities and sacrificed periodically to assess the above.** The abbreviations used are: i.p. intra-peritoneal; i.t. intra-tracheal; BAL, bronchoalveolar lavage; PB, peripheral blood.

C57Bl/6 female mice (8–10 weeks of age) were intra-tracheally instilled with 0.075 U/ml bleomycin in 40 μl volume under brief isofluorane anesthesia and animals were maintained under SPF conditions in the UW animal facilities and sacrificed periodically to below-mentioned parameters (Figure 1). Lung fibrosis, ancillary inflammatory parameters and inflammatory cell recruitment patterns were studied as follows:

#### Assessment of extent of fibrotic and inflammatory damage in the lung post bleomycin treatment

Mice were sacrificed at 10 weeks and blood, lung parenchyma enzymatically digested by dispase 1.2 U/ml and BALf (lavage volume 400 μl × 3 with ice cold PBS) were analysed. Total number of cells were assessed by Z1 Beckman Coulter particle counter. PB extrapolated to 2 ml (=volume of total peripheral blood in a 20 gm mouse), lung parenchyma of both lungs per mouse assessed and BALf was also of both lungs per mouse. Total freshly synthesized tropocollagen quantified in post-bleomycin lung of WT and gp91phox-/- show respectively a 3-folds and a 4.6-folds increase compared to their untreated or placebo-treated counterparts. DKO mice strikingly show no increase in collagen content and lung homogenate shows tropocollagen at similar levels to that of untreated. By histology and histochemical and immunohistochemical staining, lung parenchyma and areas surrounding small brochelar ducts show enhanced inflammatory recruitment, increased TGF-β secretion, and collagen deposits around small and large airways [2].

#### Inflammatory cell accumulation in the lungs and airways over time post bleomycin treatment

Tables 2, 3, 4 and 5 show significant inflammation in the lungs and airways by day 7 of bleomycin treatment. While WT post-bleomycin shows increase in total inflammatory cell number in the BALf by 7-folds over untreated, gp91phox-/- shows more than 10-folds but DKO show no appreciable inflammation neither in the untreated nor in the treated lung. Inflammatory cell components in the airways of bleomycin treated lungs in WT is mostly lymphocytes (157-fold) and neutrophils (150-fold) while macrophages increase by 2.6-fold. In the gp91phox-/- mice, however, there is a slight shift in the inflammatory cell recruitment profile where in the post-bleo lung lymphocytes (187-fold) occupy a higher share of the inflammatory exudates in proportion to the other cell types which show apportionment of 6-fold higher number of macrophages and 35-fold increase in number of neutrophils over control. Therefore in the gp91phox-/- lung the shift is in the myeloid population which shows a somewhat diminished migration to airways compared to the lymphoid population although overall inflammation is more pronounced than in the WT. DKO airways contain slightly higher number of inflammatory cells post bleo but that number is similar to the WT control and also not significantly enhanced.

**Table 2:** **Number of hematopoietic and non**-**hematopoietic cells in BALf and lung a week after bleomycin treatment**- **Trends in cellular migration to BALf and Lung post**-**bleomycin overtime**

	WT saline (d7)	WT bleo (d7)	NOX saline (d7)	NOX bleo (d7)	DRO saline (d7)	DKO bleo (d7)
Totalcellcontent (xlIYIml)	4.27 ± 0.184	28.3 ± 0224	4.515 ± 0.223	48.91* ± 1.59	2.61 ± 0.16	8.42 ± 0.13
Macrophaqes (xlIYIml)	4.16 ± 0.57 (972%)	11.14 ± 4.08 (37,6%)	4.01 ± 0.31 (89.9%)	28.14* ± 6.83 (57.47%)	1.87 ± 0.64 (71.64%)	4.19 ± 3.18 (49.82%)
Lymph ocytes (xWlml)	0.08 ± 0.01 (2.0%)	12.63 ± 2.49 (46.0%)	0.09 ± 0.02 (2.01%)	16.91 3.65ç34.53%)	1.2 ± 0.01 (4.59%)	1 .44 0.66 (17.12%)
Neutrophils (x’llYIml)	0.03 ± 0.01 (0.7%)	4.5 ± 0.97 (16.3%)	0.11 ± 0.01 (0.02%)	3.91 ± 053 (7.98%)	0.63 ± 0.14 (24.13 96)	278 ± 1.06 (33%)

Number of cells in BALf after a week of bleomycin treatment.

After the single intra-tracheal dose of bleomycin, animals were sacrificed on d7, d14, and d21 for the WT after bleo, and at D7 and d21 only for NOX-/- and DKO mice and wildtype saline treated mice. Cell number was quantitated by hemocytometer and corroborated by Z1 particle counter (Beckman Coulter). Number of cells represent data from 2 independent experiments ± SEM. N = 4/group. Cell subsets were quantified by FACS and morphologic differentiation under light microscope. Olympus BX41 at 40× magnification. Abbreviations used are: WT (wild type), NOX-/- (gp91phox knockout), gp91phox MMp12 double knockout (DKO).CD45+ cells were gated and macrophages (Gr1-F4/80hi), lymphocytes (CD3+ and B220+), and neutrophils (Gr-1hi F4/80-) were identified, their percentages analyzed by CellQuestPro and their total numbers calculated from the total number of cells obtained by lavage as described in Materials and methods. Data presented is pooled from 2 independent experiments ± SEM. *denotes p value < 0.05 compared to WT post-bleo on corresponding time points. Abbreviations used are: WS = Wildtype (WT) + saline (d7), WB7 = WT + post-bleomycin d7, WB14 = WT + post-bleomycin d14, WB21 = WT + post-bleomycin d21, NOXS = gp91phox-/- + saline d21, NOXB21 = gp91phox-/- + bleomycin d21, DKOS = gp91phox-MMP12 double knockout + saline (d21), DKOB21 = gp91phox-MMP12 double knockout + bleomycin (d21).

**Table 3:** **Number of hematopoietic and non**-**hematopoietic cells in BALf and lung a week after bleomycin treatment**- **Trends in cellular migration to BALf and Lung post**-**bleomycin over time**

BALf								
1OIml Total cells	WS 4.27 ± 018	WB7 283 ± 0.22	W614 1032 ± 2.74	W621 9.32 ± 1.74	NOXS 451 ± 0.23	NOXB2I 8.36 ± 132	DROS 4.55 ± 016	DKOS2I 5fl3 ± 1.94
Macrophage	416 ± 057	1114 ± 4.08	8.91 ± 4.93	7.06 ± 3.92	3.86 ± 1.07	6.91 ± 1.96	3.91 ± 0.74	4.03 ± 0.43
Lymphocyte	0.08 ± 0.01	12.63 ± 2.49	1.76 ± 0.67	1.33 ± 0.67	1.32 ± 0.54	2.43 ± 0.43	1.44 ± 0.32	0.56 ± 0.21
PMN	0.03 ± 0.01	4.5 ± 0.97	0.41 ± 0.07	0.87 ± 0.32	0.67 ± 0.32	1.02 ± 0.22	0.67 ± 0.11	0.41 ± 0.13

Number of CD45+ cells in BALf before and on d7, d14 and d21 after bleomycin treatment (1 i.t. dose of 0.074 U/ml in 40 μl volume).

After the single intra-tracheal dose of bleomycin, animals were sacrificed on d7, d14, and d21 for the WT after bleo, and at D7 and d21 only for NOX-/- and DKO mice and wildtype saline treated mice. Cell number was quantitated by hemocytometer and corroborated by Z1 particle counter (Beckman Coulter). Number of cells represent data from 2 independent experiments ± SEM. N = 4/group. Cell subsets were quantified by FACS and morphologic differentiation under light microscope. Olympus BX41 at 40× magnification. Abbreviations used are: WT (wild type), NOX-/- (gp91phox knockout), gp91phox MMp12 double knockout (DKO). CD45+ cells were gated and macrophages (Gr1-F4/80hi), lymphocytes (CD3+ and B220+), and neutrophils (Gr-1hi F4/80-) were identified, their percentages analyzed by CellQuestPro and their total numbers calculated from the total number of cells obtained by lavage as described in Materials and methods. Data presented is pooled from 2 independent experiments ± SEM. Abbreviations used are: WS = Wildtype (WT) + saline (d7), WB7 = WT + post-bleomycin d7, WB14 = WT + post-bleomycin d14, WB21 = WT + post-bleomycin d21, NOXS = gp91phox-/- + saline d21, NOXB21 = gp91phox-/- + bleomycin d21, DKOS = gp91phox-MMP12 double knockout + saline (d21), DKOB21 = gp91phox-MMP12 double knockout + bleomycin (d21).

**Table 4:** **Number of hematopoietic and non**-**hematopoietic cells in BALf and lung a week after bleomycin treatment**- **Trends in cellular migration to BALf and Lung post**-**bleomycin over time**

LP (d7)						
105/ml	WS	WB	NOXS	NOXB	DKOS	DKOB
Total cells	7.27 ± 0.07	38.3 ± 4.04	8.86 ± 0.08	54.19* ±2.6	6.85 ± 0.27	9.56 ± 0.65
Macrophage	6.16 ± 0.57	22.14 ± 4.08	7.13 ± 2.43	31.38* ±11.32	5.41 ± 1.43	6.36 ± 2.71
Lymphocyte	1.08 ± 0.01	9.63 ± 2.49	0.97 ± 0.12	21.41* ± 4.93	0.93 ± 0.21	2.47 ± 0.67
**PMN**	**0.03 ± 0.01**	**7.5 ± 0.97**	**0.72 ± 0.04**	**1.34 ± 0.15**	**0.51 ± 0.03**	**0.73 ± 0.33**
%	WS	WB	NOXS	NOXB	DKOS	DKOB
Macrophage	84.73 ± 1.86	57.80 ± 11.75	80.47 ± 12.9	57.96 ± 2.98	78.97 ± 3.7	66.52 ± 5.98
Lymphocyte	14.85 ± 2.84	25.14 ± 4.96	10.94 ± 1.95	39.50 ± 3.67	13.57 ± 3.98	25.83 ± 8.43
PMN	0.41 ± 0.04	19.58 ± 6.73	8.12 ± 2.07	2.47 ± 1.09	7.44 ± 1.97	7.63 ± 2.06

Number of (CD45+) crlls in LP before and after bleo treatment.

After the single intra-tracheal dose of bleomycin, animals were sacrificed on d7, d14, and d21 for the WT after bleo, and at D7 and d21 only for NOX-/- and DKO mice and wildtype saline treated mice. Cell number was quantitated by hemocytometer and corroborated by Z1 particle counter (Beckman Coulter). Number of cells represent data from 2 independent experiments ± SEM. N = 4/group. Cell subsets were quantified by FACS and morphologic differentiation under light microscope. Olympus BX41 at 40× magnification. Abbreviations used are: WT (wild type), NOX-/- (gp91phox knockout), gp91phox MMp12 double knockout (DKO). CD45+ cells were gated and macrophages (Gr1-F4/80hi), lymphocytes (CD3+ and B220+), and neutrophils (Gr-1hi F4/80-) were identified, their percentages analyzed by CellQuestPro and their total numbers calculated from the total number of cells obtained by lavage as described in Materials and methods. Data presented is pooled from 2 independent experiments ± SEM. *denotes p value < 0.05 compared to WT post-bleo on corresponding time points. Abbreviations used are: WS = Wildtype (WT) + saline (d7), WB7 = WT + post-bleomycin d7, WB14 = WT + post-bleomycin d14, WB21 = WT + post-bleomycin d21, NOXS = gp91phox-/- + saline d21, NOXB21 = gp91phox-/- + bleomycin d21, DKOS = gp91phox-MMP12 double knockout + saline (d21), DKOB21 = gp91phox-MMP12 double knockout + bleomycin (d21).

**Table 5:** **Number of hematopoietic and non**-**hematopoietic cells in BALf and lung a week after bleomycin treatment**- **Trends in cellular migration to BALf and Lung post**-**bleomycin over time**

	Saline	Bleomycin		
WT	d21	d7	d14	d21
AEI	95.7 ± 4.87	79.47 ± 2.86	67.43 ± 1.76	58.41 ± 4.83
AEII	4.3 ± 1.76	9.41 ± 1.86	8.66 ± 1.07	5.96 ± 1.12
NOX-/-				
AEI	95.7 ± 3.87	66.21±	58.23 ± 2.34	56.14 ± 3.97
AEII	4.3 ± 1.12	4.89±	5.43 ± 1.06	5.77 ± 0.56
DKO				
AEI	95.7 ± 4.16	96.23 ± 4.75	96.44 ± 11.23	97.41 ± 3.87
AEII	4.3 ± 0.56	4.5 ± 0.09	4.86 ± 1.94	4.96 ± 1.87

Number of CD45- cells in LP counterstained with alveolar epithelial cell markers before and after bleomycin treatment.

After the single intra-tracheal dose of bleomycin, animals were sacrificed on d7, d14, and d21 for the WT after bleo, and at D7 and d21 only for NOX-/- and DKO mice and wildtype saline treated mice. Cell number was quantitated by hemocytometer and corroborated by Z1 particle counter (Beckman Coulter). Number of cells represent data from 2 independent experiments ± SEM. N = 4/group. Cell subsets were quantified by FACS and morphologic differentiation under light microscope. Olympus BX41 at 40× magnification. Abbreviations used are: WT (wild type), NOX-/- (gp91phox knockout), gp91phox MMp12 double knockout (DKO). CD45+ cells were gated and macrophages (Gr1-F4/80hi), lymphocytes (CD3+ and B220+), and neutrophils (Gr-1hi F4/80-) were identified, their percentages analyzed by CellQuestPro and their total numbers calculated from the total number of cells obtained by lavage as described in Materials and methods. Data presented is pooled from 2 independent experiments ± SEM. Abbreviations used are: WS = Wildtype (WT) + saline (d7), WB7 = WT + post-bleomycin d7, WB14 = WT + post-bleomycin d14, WB21 = WT + post-bleomycin d21, NOXS = gp91phox-/- + saline d21, NOXB21 = gp91phox-/- + bleomycin d21, DKOS = gp91phox-MMP12 double knockout + saline (d21), DKOB21 = gp91phox-MMP12 double knockout + bleomycin (d21).

### Stem cell niches characterization in bleomycin-induced injury model

#### Rationale of long term assay

In a previous study, gp91phox-/- mouse had a spontaneous pro-inflammatory phenotype and then a more exaggerated emphysematous phenotype in a cigarette-smoke induced mouse model, while a DKO mouse lacking in both gp91phox and MMP12 failed to develop emphysema even after chronic cigarette smoke exposure for over a month. This model is chiefly by macrophage driven pathogenesis while an allergen-induced asthma model is Th2 cytokine driven and lymphocyte orchestrated model. In order to explore the role of oxidase and matrix metalloprotease deletion in the pathogenesis of another respiratory disease which develops through a different pathway. In other words, the aim of the study was to explore whether reactive oxygen species and matrix proteases may have a role in an allergic set up where phagocytic cells are responsible for a much downstream regulation of the inflammatory process. Further to the allergic injury model which is mainly inflammatory in nature, we wished to explore the status of regeneration in the mouse lung and thus “tease out” the lung stem cell niches to reveal themselves. Hence an injury model of bleomycin-induced idiopathic pulmonaty fibrosis was used.

#### Long term BrdU pulse chase assay

Long term assay to detect stem cell niches exposed by the bleomycin-induced injury model [52]. Marker expression analyses by FACS help identify BrdU positive cells (stem cells?) and BrdU negative cells (either accessory cells characterized by their typical surface marker expression or parenchyma other than stem cells) were assessed as percent total cells by FCM by conjugated monoclonal antibodies (Tables 6). Total number of cells remain unaltered in BM, PB, lung and BALf. Probably after 10 weeks of chase whatever temporary alteration in cells occurred at short term (1–3 weeks post-bleo) reached equilibrium at the end of 10 weeks. BrdU positive cells are the stem cells and marker expression on them identifies them. So double positive cells are stem cells and characterized by their marker expression. BrdU- cells are the rest of the cells that are also characterized by their marker expression. These are presumably the non-stem cells that may be accessory cells if present adjacent to the stem cell niches (to be corroborated by IHC by spatial distribution). The percentages shown in the datasheet are obtained by gating on BrdU positive or negative cells.

**Table 6:** **Percent positive cells by FACS within BrdU** + **and BrdU**- **population in lung for pluripotent and pulmonary lineage specific markers**

	BrdU-	TTF-1+	Oct3/4+	SSEA-3+	SSEA-4+	Sca-1+	Lin-	CD34+	CD31+	SP-C+	AQP-5+	CC-10	CD45+	CD45-	SP-C + CC10+	VEGF
Con 2d	92.13 ± 0.84	0.38 ± 0.10	0.560.09	0.55 ± 0.02	0.47 ± 0.14	0.22 ± 0.15	0.2 ± 0.01	0.31 ± 0.06	0.03 ± 0.01	5.59 ± 0.11	90.92 ± 0.50	1.30 ± 0.18	2.37 ± 0.17	83.70 ± 1.66	0.03 ± 0.01	2.86 ± 0.09
Con 6d	92.95±	0.31 ± 0.07	0.35 ± 0.02	0.4 ± 0.07	0.32 ± 0.03	0.06 ± 0.02	0.19 ± 0.04	0.22 ± 0.009	0.22 ± 0.09	4.90 ± 0.14	85.75 ± 2.21	1.33 ± 0.21	2.7 ± 0.16	76.15 ± 1.69	0.12 ± 0.03	2.31 ± 0.14
Bleo 2d	89.06 ± 0.66	11.36 ± 0.59	1.11 ± 0.09	0.28 ± 0.07	0.35 ± 0.02	0.12 ± 0.07	0.34 ± 0.03	3.45 ± 0.09	0.71 ± 0.042	1.48 ± 0.17	9.29 ± 0.41	2.60 ± 0.16	0.78 ± 0.06	74.65 ± 1.37	0.01 ± 0.004	12.74 ± 0.59
Bleo 4d	89.43 ± 0.66	12.13 ± 0.74	1.34 ± 0.09	0.32 ± 0.04	0.26 ± 0.08	0.25 ± 0.03	0.24 ± 0.08	3.42 ± 0.23	0.8 ± 0.09	1.74 ± 0.03	217.49 ± 208.50	2.85 ± 0.08	0.65 ± 0.13	*74.65* ± *1.37*	0.02 ± 0.004	12.45 ± 0.84
Bleo 6d	97.40 ± 1.1	11.68 ± 0.6	1.742 ± 0.16	0.31 ± 0.04	0.32 ± 0.01	0.16 ± 0.05	0.54 ± 0.05	2.90 ± 0.06	0.6 ± 0.086	1.52 ± 0.06	9.05 ± 0.18	2.36 ± 0.19	0.81 ± 0.13	83.70 ± 1.66	0.12 ± 0.06	10.65 ± 0.26

BrdU + cells were gated and inside (positive and outside of the gate, BrdU negative) cells were characterized by FACS with fluorochrome conjugated monoclonal antibodies markers of pluripotence (TTF-1, SSEA-3,4, Oct ¾, SCA-1, Lineage negative and CD31 (endothelial progenitor) and CD34 (hematopoietic progenitor). lung lineages are characterized by the following markers: AQP-5+ cells are AEI, SP-C + cells are AEII and CC-10+ cells are Clara cells while SP-c + CC-10+ cells are broncho-alveolar stem cells.

### Detection of stem cells by CFU-C

BrdU single positive cells in the lung parenchyma was decreased by 4.8-fold post-bleo. So putative stem cell population seem to be gradually depleted from lung upon bleo treatment. 0.1×10^6^ cells were plated in semi-solid methyl cellulose and CFU counted after 14 days [23].

### Detection of stem cells by marker expression

#### Characterization of BrdU + cells

Only BrdU + cells were sorted and plated as these are the slow cycling stem cells derived from the lung. Marker expression was studied by FACS analysis to characterize stem and mature differentiated cells (Table 6). TTF-1 which is a typical expression marker of AEII, have been implicated with early lung development. Post-bleo, there was 4.9-folds decrease in these cells showing that putative progenitors are destroyed. Oct3/4 is a early pluripotent marker. Post-bleo there is 1-fold decrease in Oct3/4 + cell percent among BrdU + cells. This may indicate a universal downregulation of pluripotency post-bleo and may be due to an overall depletion of the stem cell reserve or individual cells may lose Oct3/4 expression in limited pockets (calculation of kd value by Scatchard analysis may give a more exhaustive quantitative assessment). SSEA-3 and 4 were decreased by 5.9-fold and 6.14-fold respectively post-bleo compared to control. Since these are stem cell specific antigens, this may again point towards an overall depletion of pluripotent reserve cells in niches. Sca-1+ cells are mouse specific stem cells. Being BrdU + these are undoubtably stem cells in the lung. 22.6-fold decrease in Sca-1-BrdU double positive cells may indicate again that post-bleo progenitor population decreased in lung. Lineage negative cells are those that are non-hematopoietic in origin and here, being BrDU positive are the definitively non-hematopoietic cells in lung that have stem like properties. 2.6-fold decrease in these cells in bleo treated mouse lungs indicate that stem cells of non-hematopoietic origin are depleted. CD34+ cells are hematopoitic cells and being BrdU + must have stem like properties. 2.2-fold increase in these cells post-bleo indicate that hematopoietic stem cells definitely have an impact post-bleo on lung stem cell content. Our theory is that either hematopoietic stem cells from bone marrow or other tissues may travel to injured lung to replenish depleted lung stem cell niche. CD31+ cells are endothelial progenitors. 1.9-fold increase in these cells which are also BrdU positive. SP-C + are AEII cells. There was 14.3-fold decrease post-bleo. AEII are traditionally known progenitors who are assumed to trans-differentiate to AEI upon bleo-induced depletion of the former. Being BrdU + here they are conclusively slow cycling cells although expressing SP-C, a marker of mature AEII cells. This is interesting because decrease in this double positive population shows either that pre-destined SP-C + LRC are depleted post-bleo or that SP-C positive cells which revoke their progenitor property and become slow cycling, probably as a preamble to trans-differentiation into AEI, become less and less. AQP-5+ AEI cells were quite low in percent to start with and became even less post-bleo. However, as a technical note, since the percent positive cells were extremely low to stat with (control), the 6-fold decrease post-bleo may not be significant. Some Clara cells have also been traditionally known to possess stem like properties (for instance, naphthalene resistance). Here CC-10+ BrdU + cells increased by 1.2-fold post-bleo showing that Clara cells probably have some definite responsive function to bleo challenge.

#### Characterization of Non-stem cells (BrdU-)

These are the normally cycling cells presumably the other cells constituting the rest of the lung parenchyma. There was no significant difference between pre- and post-bleo BrdU- population in mouse lung. BrdU + and BrdU negative cells showed expected relative distribution in the same scattergram making a 100%. TTF-1+ BrdU- cells were decreased by 36.78-fold. Since TTF-1 is co-expressed on AEII, increase in this cell population may indicate an alteration in AEII response to bleo. Whether this has any significance to stem cell niche in lung is unknown. Oct3/4 + BrdU- cells were decreased by 4.97-fold post bleo. So along with the stem cell population, the mature cells were also probably universally depleted to be replaced by collagen fiber. Neither SSEA-3 nor 4 nor Sca-1+ cells show any difference in percentage before and after bleo when gated on BrdU- cells. These markers are expressed on extremely low number of cells and hence may be of no consequence in this response. Lineage- cells show a statistically significant 2.8-fold increase. Again this may indicate that non-hematopoietic cells in the lung are universally depleted post-bleo. Again the percentage of this population is so low that this may be technically ignored.

CD34+ hematopoietic progenitors which were not LRC were also concommittantly increased by 14.5-fold post bleo again corroborating our hypothesis that there is a recruitment of progenitors of hematopoietic origin in response to bleo. Endothelial progenitors CD31+ cells were also increased by 2.9-folds post-bleo. These are the non-stem CD31+ in lung. We do not know how this may be significant. However, again the cell percent is extremely low. SP-C + AEII were decrased by 3.2-fold and AQP-5+ AEI by 9.5-fold. This is in keeping with the overall concept of degeneration of alveolar epithelium in bleo-induced fibrosis. Clara cells were decreased by 1.3-fold and is statistically significant. CD45+ and CD45– cells were decreased by 3.3-fold and 1.1-fold post-bleo again in keeping with the overall degeneration of lung cell parenchyma in bleo-induced fibrosis.

### Short term hoechst SP experiment

#### Rationale of short term assay

Stem cells are known to efflux Hoechst dye very slowly and so when flow sorted at 90 min post Hoechst incubation, cells that efflux the dye are gated and sorted as the Side population cells (SP) that are assumed to be the lung stem cells. Previously most SP studies were done on bone marrow derived cells. To our knowledge, there is no publication to date about sorting and *ex vivo* culture of SP cells from lung. Sp ours is the first study of this nature where before and after bleo (7 days after single i.t instillation of 0.075 U/ml bleomycin) in C57Bl/6 mice of three genotypes wildtype (WT), gp91phox-/- (NOX-/-), and MMP12-gp91phox double knockout, lungs were digested with dispase 1.2 U/ml in 37°C for an hour and then incubated with 1:200 from Hoechst stck (aliquoted and frozen at -20°C in 37°C water bath for 90 min and then flow sorted on FACSAria gating on a small (0.1%) cell population forming the characteristic “shoulder” of actively dye-effluxing cells. These cells were then plated in mouse ES medium + LIF and characterized by surface marker expression through 5 days of culture and then frozen in straws in liquid nitrogen. WS = WT + saline, WB = WT + bleo etc.

#### Analysis of inflammatory cells post bleomycin treatment in single and double knockout mice

Cellularity of lung parenchyma was measured in single cell suspension by Z1 Beckman particle counter. Only lung parenchyma of d7 post bleo were considered as our purpose is to look for stem cells in the lung and d7 post bleo is when the changes in cell number are most apparent then by d21, cell number of neither BALf not lung show any significant difference between before and after bleo treatment groups and it is the histopathologic assessment of the lung that shows conclusive proof of the fibrotic process having occurred in the lung [23].

### Cells in BALf and lung post bleomycin over time

Total number of cells and cell subsets was measured periodically over time in WT, and the KO mice (Tables 2, 3, 4 and 5). Total cells was increased in bleomycin-treated NOX-/- mice at d7 after bleo with macrophages and lymphocytes contributing the most of the increase. PMNs also increased in percentage but total number was comparable to that in post-bleo WT. DKO on the other hand did not show very low increase in total number of cells over their saline treated control group with lymphocytes and PMNs contributing to the slight increase entirely. Compared to saline treated WT, number of cells increased 6.6-folds a week after bleomycin treatment. This denuded at d14 and further at d21. So similar to previous data, cell number in BALf is actually not an indicator of the extent of fibrosis by d21. Similar trends were found in lung (Table 4). D7 probably signals onset of fibrosis by increased cellular recruitment. Macrophages and T cells are the chief secretors of TGFβ traditionally thought to activate collagen synthesis and deposition by alveolar epithelial cells. d8-21 therefore is the scar tissue formation period when AEI have become denuded and so have AEII. This is shown in Table 5 where there is a progressive decrease in AEI through d21 while AEII first increased slightly only to equilibriate at d21. NOX-/- BALf showed a 1.7-fold increase in cell number which decreases predictably by d21. DKO BALf however shows no appreciable increase over saline treated either at d7 or at d21. Macrophages seem to be the chief cell populations accounting for this increase. In lung (Table 4) however, on d7, a similar trend to that in BALf is found. The only difference is that both macrophages and lymphocytes make up for the increase.

### Characterization of sorted SP cells in *ex vivo* culture

Sorted cells from day 7 post bleo mice and their corresponding control groups from all three genotype groups were plated in mouse ES and FACScan of surface expression markers evaluated every 24 h in culture before freezing on d5 of culture. So data represents percent positive for each marker expression on flow sorted SP cells in *ex vivo* culture with LIF.

In the bleomycin induced fibrosis experiments done earlier and repeated with this latest batch of mice, it is apparent that while gp91phox-/- mice respond with an exaggerated fibrotic manifestation in lung and MMP12- gp91phox double knockout (DKO) mice show little or no change from their saline treated counterparts. Isolation of SP cells from the lungs of these animals on d7 post-bleomycin and ex vivo culture for 5 days may explain the mechanism for such response on the basis of changes in their marker expression in culture with LIF (to not promote differentiation).

Overall, SSEA-3 and 4 remained stationary at quite high expression throughout culture period with cells both from untreated lung cells from all three genotype groups as well as bleomycin treated WT lung except the two knockout mice post bleomycin where both are significantly downregulated. Other pluripotent marker expression was downregulated in all three post-bleo but of note is the different expression of SP-C-CC-10 double positive cell population in both saline treated and bleomycin treated knockout cells. The significance of this difference is unknown.

### Discussion

SP cells are believed to be derived from the bone marrow and can be differentiated from committed tissue stem cells. In models of ablative radiation injury, CD45+ lung SP cells have demonstrated transient repopulation of the bone marrow and indicated that CD45+ lung SP cells are analogous to the short-term repopulating hematopoietic cell. In models of ablative radiation injury, CD45+ SP cells have been demonstrated to be sufficient for reconstitution of the bone marrow [9, 32]. In addition, in one report, marked SP cells were shown to repopulate damaged lung [53] in irradiated mice, resulting in rare, but detectable, fibroblasts and alveolar and bronchial epithelial cells. These findings support the position that tissue SP cells are hematopoietically derived, pluripotent stem cells that may play an important role in tissue repair. Other studies have attempted to address whether SP cells that are localized to specific tissues maintain their pluripotency. Distinct populations of tissue-localized SP or Lin–, Sca-1+ cells that maintain hematopoietic activity have been identified in muscle [54, 55] and liver [40]. Interestingly, these SP cells have not been identified in peripheral blood and are least frequently found in the bone marrow (0.79% of nucleated cells) compared with other tissues (0.96–15.1% of nucleated cells) [56].

It is generally important to elucidate airway epithelial cell lineages and to identify multipotent progenitors as targets for gene therapy. Stem (S) cells are typically present in specialized compartments spatially proximal to their differentiated progeny, but an equivalent paradigm has not been demonstrated in the airway. We discovered a distinct population of cells displaying high levels of keratin expression in murine tracheal submucosal gland ducts, and tested the hypothesis that bromodeoxyuridine (BrdU) label-retaining cells (LRCs), thought to represent the S-cells, were present in this compartment. Mice received weekly epithelial damage by intratracheal detergent or SO_2_ inhalation for 4 wk and received intraperitoneal injections of BrdU every 48 h during the injury and repair period. At 3 and 6 d after injury, BrdU-positive epithelial cells were noted along the entire tracheal length in both basal and lumenal cell positions. At later time points (20 and 95 d) LRCs were localized to gland ducts in the upper trachea and to systematically arrayed foci in the lower trachea, typically near the cartilage-intercartilage junction. LRCs were not pulmonary neuroendocrine cells. Heterotopic tracheal grafts after surface epithelial removal demonstrated reconstitution of a surface-like epithelium from gland remnants. These results suggest that airway epithelial S cells are localized to specific niches [57].

Bleomycin-induced idiopathic pulmonary fibrosis model in mice, WT as well as knockout, was developed in a systematic study to investigate whether inflammation may be involved down- or up-stream of the onset of the fibrotic process as well as to “tease” ot the lung stem cell niches by by inflicting “injury” to the tissue. To identify and characterize the “stemness” of the mobilized or “homing” progenitors short term assays ([36] (Table 6) and short term assays [Ray Banerjee, E. revealed that post bleomycin treatment, there was heavy inflammation in the lung parenchyma as well as airways) (Tables 2, 3, 4 and 5) and by long term assays (Table 4). The presence of a large number of myeloid inflammatory cells recruited to the airways and obtained in the exudates, shows this inflammation to be mainly Th1-driven.

As communicated in our publication [39] both inflammation and airway hyperreactivity were more extensive than in wildtype mice post-OVA in NOX or gp91phox KO mice. Although OVA-specific IgE in plasma were comparable in wildtype and knockout mice, enhanced inflammatory cell recruitment from circulation and cytokine release in lung and BALf, accompanied by higher airway resistance as well as Penh in response to methacholine, indicate a regulatory role for NADPH oxidase in development of allergic asthma. Also, while T cell mediated functions like Th2 cytokine secretion, and proliferation to OVA were upregulated synchronous with the overall robustness of the asthma phenotype, macrophage upregulation in functions such as proliferation, mixed lymphocyte reaction, and MCP-1 directed chemotaxis, but downregulation of respiratory burst response indicate a forking in their signaling pathways. gp91^*phox*^-/- MMP12 double knockout (DKO) mice show a similar phenotype as the gp91^*phox*^-/- showing the non-involvement or synergistic involvement of MMP12 in the response pathway. Contrary to a Th2-driven inflammation as in this OVA-induced allergic asthma model, bleomycin-induced IPF obviously takes a different afferent immune response pathway to develop. However, the most striking finding is with the fibrosis model, where there is no inflammation in the double knockout mice as well as no fibrosis indicating that inflammation is a pre-requisite to fibrosis.

BrdU + CD45+ population are hematopoietic cells with pluripotent properties. Post bleo they are increased by 0.7 fold again corroborating our theory that progenitors from circulation forms is a component of the lung stem cell scenario. Most BrdU + cells were CD45- cells (non-hematopoietic origin). There was no significant alteration in their number when assessed by gating on CD45- alone. SP-C + CC-10+ cells are the BASCs (broncho-alveolar stem cells). Gated BrdU + cells show a 38.9-fold decrease in this population which formed majority of the BrdU + cells before and constituted a bare 2% post-bleo in the mouse lung. This is perhaps the most important finding of the pulse chase study that BASCs are so drastically mobilized and depleted after 10 weeks of bleo-induced fibrosis. VEGF is a growth factor traditionally associated with increased vascularizxation. Since fibrosis is a degenerative disease, we measured VEGF + cells in the BrdU + population and found that there was 1.8-fold decrease in post-bleo. If VEGF is assumed to be a pro-fibrotic signal, then decrease in VEGF + LRC may indicate either that the fibrotic process is slowing wearing off or if VEGF is assumed to be a signal for progenitor mobilization. Of significance is that there is no change in BrdU- BASC population. Does this mean that they is a very small population of these cells which are quiescent even though the there IS a BrdU + BASc which are other wise responsive to bleo challenge. VEGF + cells were increased by 4.6-fold post bleo. Interestingly while the VEGF + LRC were decreased, VEGF + BrdU- cells were increased post-bleo. This may help counterbalance the mobilization of VEGF + cells from stem cell niche (Tables 6). There was no significant difference in colony forming potential of BrdU- cells before and after bleo post 10 week chase.

SP cells were flowsorted from lungs of mice at d7 post bleomycin exposure. This was done primarily on the assumption that the fibrosis process was just beginning. However, as apparent from cell traffic data in airways (Tables 2, 3 and 4), specially the real time trend on day 7, 14 and 21 speak of a primary inflammatory recruitment followed by an exodus out of the lung that is slowly replaced by fibrosis. This is significant. SP cells from d7 therefore are the progenitors which are mobilized coinciding with the inflammatory cell mobilization from circulation. It may be that inflammatory cells may produce signals to induce mobilization of lung stem cells from tissue niche or they may bring with them progenitors mobilized from the bone marrow. Whatever the origin, lineage commitment may be occurring when the same cells cultured *in vitro* show up- or down-regulation of different markers.

To begin their life outside the lungs, the sorted SP cells expressing mature cell markers are very low but as culture progresses in the presence of LIF (under negative selection pressure where differentiation is inhibited) markers of “stemness” get downregulated indicating that despite presence of LIF a slow maturation process has begun. Of note is the expression of the Oct3/4 pluripotent marker which is about 16% (< p 0.05) less than in the NOX compared to saline treated WT as well as saline treated DKO is of significance. This unexpected down-modulation may account for the unregulated development of fibrosis in the single knockout (gp91phox-/-) only as opposed to the DKO which is protected. Whether the MMP12 deletion in addition to the NADPH oxidase sub unit deletion has a counter and therefore protective effect towards development of fibrosis is speculative as it is beyond the scope of this paper to elaborate on it and needs more work with the Wnt/β catenin pathway to reveal molecular mechanisms underlying this protection. The extent of decrease in its expression in the bleomycin treated cells is also 2-folds greater in the gp91phox-/- compared to WT and DKO mice derived cells. The other interesting modulation in marker expression is that of the double positive BASC cells (SP-C + CC-10+) that is sharply down-regulated by the d2 of culture in the WT but a much more gradual down-regulation in the gp91phox-/- (d4). The DKO on the other hand is 2-fold less than the first two, in the saline treated and in the bleomycin treated while percentage of these BASC in the WT and gp91phox-/- go down to almost nil (since this is an ex vivo culture excluding cell turnover per se, we may assume that the markers themselves downregulate. In the absence of more detailed study, we may simply comment that the rate and degree of BASC (broncho-alveolar stem cells) may have a developmental significance in the progression of fibrosis. Mesenchymal cells as well stroma of the lungs may have a significant role to play in the future development of the cells [30, 36, 58].

In the end, as a commentary and hypothesis, we offer the following views:

The cells within a tissue such as the lung where rapid turnover of cells is the rule where efficient regeneration need to balance the high level of oxidative wear and tear, we believe, in the light of the data presented in this paper, that identity of a cell, both structural and functional, undergo a dynamic plasticity by which they can change back and forth between functionally competent lineage restricted cells of the different anatomical tissue sub-types of the lung and progenitor-like cells that can differentiate and trans-differentiate into cells of the required type according to the current demand to restore homeostatsis in the lung [9, 30, 32]. To accomplish this there must be efficient and seamless de-differentiation and differentiation of the same cellular pool by simply switching on and off, certain defined molecular switches. The data showing dynamics of marker expression in lung and airways of an injured lung in real time testify to this. The so-called “niches” therefore m ay be both anatomical, occupying distinct regions of the lung, as well as functional, that is irrespective of their spatial distribution, cells can undergo rapid changes as to their developmental identity according to the exigent situation born out of a particular injury.

### Conclusion

In summary, (A) from the long term BrdU pulse chase experiments the following inferences can be drawn: (1) The increase in the hematopoietic progenitor pool in lung indicated that exogenous progenitors from circulation contribute to lung regeneration; (2) most non-stem cells (accessory cells?) were non-hematopoietic in origin indicating that despite tissue turnover and some spontaneous resolution of fibrosis as reported, BASCs are drastically depleted possibly necessitating recruitment of progenitors from hematopoietic pool; (3) loss of VEGF + LRC may indicate either a gradual resolution of the fibrosis process or a signal for progenitor mobilization from niches; (4) BrdU negative BASC population may be a small quiescent population that remains as a reserve for more severe lung injury; (5) increase in VEGF + non-LRC may indicate a checkpoint to counterbalance the mobilization of VEGF + cells from the stem cell niche and a protective measure against complete depletion of the same. (B) From the short term SP population analyses studies, the following conclusions can be drawn: (6) Inflammation is a pre-requisite for fibrosis; (7) SP cells, being the putative stem cells in the lungs, were increased (either by self renewal or by recruitment from the exogenous bone marrow pool) post-bleomycin in NOX-/- but not in DKO indicating the necessity of cross-talk between gp91phox and MMP-12 in this process; (8) *ex vivo* cultured SP progressively lose pluripotent markers, notably BASC (SPC + CC10+) - significance is unknown.

## Abbreviations

FACS: Fluorescence activated cell sorting
MACS: Magnetic activated cell sorting
HSCs: Hematopoietic stem cells
MSCs: Mesenchymal stem cells
SDF – 1: Stromal derived factor – 1
ESCs: Embryonic stem cells
iPS cells: Induced pluripotent stem cells
ICM: Inner cell mass
PBS: Peripheral blood stem cells
GVHD: Graft versus host disease
GSCs: Germline stem cells
fGSCs: Female germline stem cells
mGSCs: Male germline stem cells
ISCs: Intestinal stem cells
FSCs: Follicle stem cells
ESCs: Escort stem cells
NB: Neuroblast
GMC: Ganglion mother cell
HP: Hematopoietic precursor
PSC: Posterior signaling center
MT: Malpighian tubules
RNSCs: Renal and nephric stem cells
ECM: Extracellular matrix.
